# 1077. Understanding the Psychosocial Burden Associated with Hospitalization Among Adults Diagnosed with COVID-19 in the United States

**DOI:** 10.1093/ofid/ofac492.918

**Published:** 2022-12-15

**Authors:** Wajeeha Ansari, Florin Draica, Joanna Atkinson, Kathy Annunziata, Martine C Maculaitis, Amie Scott

**Affiliations:** Pfizer Inc., Wyckoff, New Jersey; Pfizer, Inc., New York, New York; Pfizer Inc., Wyckoff, New Jersey; Cerner Enviza, Malvern, Pennsylvania; Cerner Enviza, Malvern, Pennsylvania; Pfizer Inc., Wyckoff, New Jersey

## Abstract

**Background:**

Due to the coronavirus disease 2019 (COVID-19) pandemic in the United States (US), public health officials sought to reduce transmission. However, the psychosocial impact associated with COVID-19 has received less attention. This study describes psychosocial burden among adults diagnosed with COVID-19 and assesses the unique impact on those who had a COVID-19 hospitalization.

**Figure:**
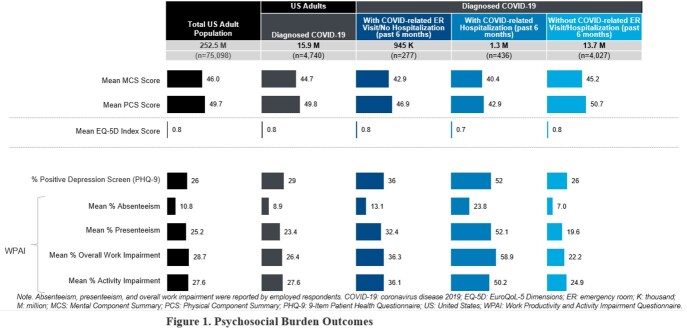

**Methods:**

This cross-sectional retrospective study used 2021 US National Health and Wellness Survey (NHWS; N=75,098) data. NHWS is an annual web-based self-report survey of the US general adult population (aged ≥ 18 years). Results were weighted to reflect the population on age, gender, race/ethnicity, and education based on US Census. Among adults who self-reported a COVID-19 diagnosis, those with COVID-related hospitalization, emergency room (ER) visit/no hospitalization, and no hospitalization/no ER visit were descriptively compared on demographics, health characteristics, and psychosocial burden measures.

**Results:**

Almost 16 million adults had a COVID-19 diagnosis in the past year; of these, 8% had a COVID-related hospitalization, and 6% had a COVID-related ER visit/no hospitalization. Compared to adults with no ER visit/no hospitalization or ER visit/no hospitalization, those with a hospitalization were more often male, college educated, and employed. Relative to those with no ER visit/no hospitalization, adults with a hospitalization were more often diagnosed, either pre- or post-COVID-19 diagnosis, with allergies (47% vs 38%), asthma (20% vs 11%), pain (37% vs. 25%), headache (25% vs 16%), migraine (27% vs 15%), type 2 diabetes (16% vs 10%), dry eye (25% vs 12%), and sleep apnea (15% vs 11%). Adults with a hospitalization had lower mental, physical, and general health-related quality of life, 2-3.4 times higher work/non-work impairment, and 2 times higher positive depression screen rate than those with no ER visit/no hospitalization.

**Conclusion:**

US adults with a COVID-related hospitalization had higher psychosocial burden than those without a hospitalization on several domains. Accordingly, reducing COVID-related hospitalizations, particularly among the employed and those with comorbidities, will be vital to help mitigate this burden.

**Disclosures:**

**Wajeeha Ansari, MPH**, Pfizer Inc.: Stocks/Bonds **Florin Draica, MD**, Pfizer Inc.: Stocks/Bonds **Joanna Atkinson, MD**, Pfizer Inc.: Stocks/Bonds **Kathy Annunziata, MA**, Pfizer: Advisor/Consultant|Pfizer: Employee of Cerner Enviza, which received funding from Pfizer to conduct and report on the study **Martine C. Maculaitis, PhD**, Cerner Enviza: Employee of Cerner Enviza, which received funding from Pfizer to conduct and report on the study. **Amie Scott, MPH**, Pfizer Inc.: Stocks/Bonds.

